# Improved Biomass Production and Secondary Metabolism: A Critical Review of Grafting in *Cannabis sativa*

**DOI:** 10.3390/plants14152347

**Published:** 2025-07-30

**Authors:** S. M. Ahsan, Md. Injamum-Ul-Hoque, Md. Mezanur Rahman, Sang-Mo Kang, In-Jung Lee, Hyong Woo Choi

**Affiliations:** 1 Department of Plant Medicals, Andong National University, Andong 36729, Republic of Korea; 2 Department of Agriculture, Gopalganj Science and Technology University, Gopalganj 8100, Bangladesh; 3 Department of Applied Biosciences, Kyungpook National University, Daegu 41566, Republic of Korea; 4 Institute of Genomics for Crop Abiotic Stress Tolerance, Department of Plant and Soil Science, Texas Tech University, Lubbock, TX 79409, USA; 5 Institute of Agricultural Science and Technology, Kyungpook National University, Daegu 41566, Republic of Korea; 6 Institute of Cannabis Biotechnology, Andong National University, Andong 36729, Republic of Korea

**Keywords:** cannabis, secondary metabolites, vascular tissue, phytohormones, oxidative stress

## Abstract

*Cannabis sativa* L. is a versatile plant with applications in various sectors such as agriculture, medicine, food, and cosmetics. The therapeutic properties of cannabis are often linked to its secondary compounds. The worldwide cannabis market is undergoing swift changes due to varying legal frameworks. Medicinal cannabis (as a heterozygous and dioecious species) is distinct from most annual crops grown in controlled environments, typically propagated through stem cutting rather than seeds to ensure genetic uniformity. Consequently, as with any commercially cultivated crop, biomass yield plays a crucial role in overall productivity. The key factors involved in cultivation conditions, such as successful root establishment, stress tolerance, and the production cycle duration, are critical for safeguarding, improving, and optimizing plant yield. Grafting is a long-established horticultural practice that mechanically joins the scion and rootstock of distinct genetic origins by merging their vascular systems. This approach can mitigate undesirable traits by leveraging the strengths of particular plants, proving beneficial to various applications. Grafting is not used commercially in *Cannabis*. Only three very recent investigations suggest that grafting holds significant promise for enhancing both the agronomic and medicinal potential of *Cannabis*. This review critically examines the latest advancements in cannabis grafting and explores prospects for improving biomass (stem, root, flower, etc.) yield and secondary metabolite production.

## 1. Introduction

*Cannabis sativa* L. belongs to the “Cannabaceae” family and is an extensively utilized plant in various sectors such as agriculture, medicine, food, and cosmetics. Medical cannabis and hemp (*Cannabis sativa* L.) contain bioactive compounds with biological effects on the human body, making them influential in the pharmaceutical industry. The medicinal properties of cannabis are mainly due to its secondary compounds, particularly terpenoids, sterols, flavonoids, and phytocannabinoids (cannabinoids), primarily found in the flowers and found in minor quantities in the leaves, stems, and other parts of the plant [1,2,3,4,5]. *Cannabis sativa* is recognized as a single species with several subdivisions. One classification is based on chemotype, distinguishing varieties, such as high tetrahydrocannabinol (high-THC, THC-dominant), high-cannabidiol (CBD-dominant), or balanced chemotypes (intermediate THC and CBD levels). In addition to THC and CBD, scientists are exploring the pharmacological possibilities of minor cannabinoids, such as “cannabigerol (CBG) and cannabichromene (CBC)” [4,5]. Though these compounds are present in low concentrations, specific strains with higher levels have been identified, and their medicinal potential is under evaluation. As clinical and pharmacological research advances, minor cannabinoids are expected to gain prominence as therapeutic targets [6,7]. The international cannabis market is experiencing rapid growth due to evolving lawful frameworks, with projected revenues of USD 61 billion in 2024 and USD 73 billion in 2027 [7,8,9]. However, as a relatively young industry, it lacks well-established agronomic and agrotechnical practices [7]. In recent years, ground breaking investigations proved that this variation in secondary metabolite profiles production is largely influenced by environmental factors and management practices, including biotic and abiotic stresses, light, altitude, mineral nutrition, nutritional availability, and the complex interplay between plant morphology, architecture, biomass, and flowering patterns, all of which serve as major determinants of phytochemical expression [1,10,11,12,13,14,15,16,17]. Although cannabis has been used historically for centuries, the comprehensive understanding of its chemical and physiological characteristics remains limited, largely due to longstanding legal constraints [10]. In several countries, legislation is changing due to the recognition of the medicinal and agricultural value of cannabis plants [10].

*Cannabis* bioactive compounds are primarily produced by unfertilised female plants, which are exclusively cultivated for secondary metabolite production. High-quality cannabis aims for consistent and uniform inflorescence yields while maximizing metabolite synthesis [7,18,19,20]. Commercial producers primarily use clonal propagation via stem cuttings rather than feminized seeds to achieve genetic uniformity, which can produce heterogeneous offspring [19,20,21]. Successful vegetative propagation is subject to factors such as “temperature, relative humidity, shoot length and thickness, and the genotype and the condition of the mother plant”. Some genotypes, however, exhibit poor rooting ability, limiting their propagation through cuttings [19]. Given the high energy demands of indoor cannabis cultivation, optimizing propagation efficiency is crucial [19,20,22]. Most commercial growers aim for at least five production cycles annually, making rapid maturation essential for maximizing output [20]. Improvements in yield can be achieved by optimizing biomass production, secondary metabolite concentration, plant density, and growth cycle duration [20].

Grafting with clonally propagated germplasm differs from traditional vegetative propagation with cuttings and has not been studied in detail in cannabis [19,23,24]. Grafting is not standardized in the cannabis industry, which mainly uses root cuttings, tissue culture plantlets, or seeds [7,19,20]. When implementing a novel methodology in horticulture, it is essential to consider specific plant traits in addition to cost considerations. In cannabis cultivation, the desirable qualities of the scion are associated with reproductive characteristics, including the duration of growth under floral induction until harvest, as well as the visual and biochemical properties of the floral organs. Additionally, crop yield is determined by the number of inflorescences per plant, their size, and total weight [7,19,20]. Grafting onto tolerant rootstocks offers significant potential to enhance water and nutrient use efficiency, promote root development, improve photosynthetic performance and hormone signalling, and ultimately mitigate yield losses while enhancing crop quality and stress tolerance [25,26,27]. Desired rootstock properties are common to most plants and are related to vegetative characteristics, such as rooting ability, mineral and water absorption, disease and pest resistance, and tolerance to abiotic stresses [7,19,20].

Even though there is growing interest in cannabis cultivation techniques, research on grafting in cannabis production remains limited, particularly regarding effects on plant morphological traits, yield, and concentrations of secondary metabolites. This gap highlights the need for comprehensive studies on grafting efficacy in cannabis. Research on various plant species has shown that grafting can alter secondary metabolism, resulting in the increased production of different bioactive compounds. The unique physiological and biochemical characteristics of cannabis necessitate focused research to explore how various combinations of rootstock and scion can prompt the production of critical medicinal compounds, especially phytocannabinoids. This review critically examines the recent advancements in cannabis grafting and explores prospects for improving biomass yield and metabolite production, identifies existing knowledge gaps, and offers insights that could lead to significant progress in medical cannabis.

## 2. Literature Search Strategy

This review synthesizes recent advances in grafting techniques aimed at enhancing *Cannabis sativa* cultivation. Specifically, it explores the influence of grafting on biomass production and metabolic processes in *C. sativa*, offering valuable insights into its practical applications. A comprehensive literature search was conducted using multiple academic databases, including Google Scholar, Web of Science (WoS), ScienceDirect, EBSCOhost, PubMed, and Scopus. The search strategy applied no publication year restrictions and focused exclusively on peer-reviewed English-language articles. The keywords used for retrieving relevant studies included “grafting”, “*Cannabis sativa*”, “rootstock”, “scion”, “metabolism”, and “biomass”.

## 3. Recent Advances in Grafting-Mediated Alteration in Biomass and Secondary Metabolism in *Cannabis sativa*

Among commercial cannabis varieties, some high-yielding cultivars have relatively weak root systems. To address this limitation, one study revealed that grafting a high-THC variety, “Freud Super-Ego” (FSE), onto three chemo-typically distinct rootstocks, such as those with high-THC, high-CBD, and balanced chemotypes, displayed significantly greater root biomass compared to FSE [7]. The female floral trichomes, where the cannabinoids and terpenes are produced, represent the primary carbon sink in cannabis plants, suggesting that rootstock selection can ultimately influence the vegetative growth of scions by enhancing the uptake of water and mineral nutrients and supporting the accumulation of biomass and cannabinoid biosynthesis [7]. Rootstock selection fundamentally alters inflorescence size and quality and influences overall market value [7]. Increased leaf production provides a greater photosynthetic surface area, enhancing carbohydrate synthesis and precursor availability for cannabinoid biosynthesis in glandular trichomes [7]. By aligning scions with compatible rootstocks, cultivators can regulate plant vigour, increase yield, and influence cannabinoid profiles [7]. Further research is needed to reveal the mechanisms governing rootstock–scion interactions and develop grafting tactics that expand crop productivity and medicinal efficacy.

Grafting in medicinal cannabis presents opportunities for exploring long-distance signalling pathways involving hormones, proteins, and RNAs [20]. Strigolactones, root-derived phytohormones, vigorously transported within the plant, play a crucial role in regulating shoot architecture, particularly lateral branching, in response to the availability of nutrients [20]. Differences in strigolactone biosynthesis, perception, and crosstalk with other phytohormones may partially clarify the morphological pattern of branching and stunting phenotypes observed in grafting processes [20]. These findings indicate that grafting could be the best alternative to enhance stress adaptation and disease resistance in medicinal cannabis by modulating hormone-induced signalling pathways [20]. Phytohormonal crosstalk is central to plant defence response, mediating secondary metabolite production under different stress conditions [28,29]. Despite the extensive characterization of cannabinoids and terpenes, relatively little attention has been paid to hormonal regulation in cannabis.

**Figure 1:**
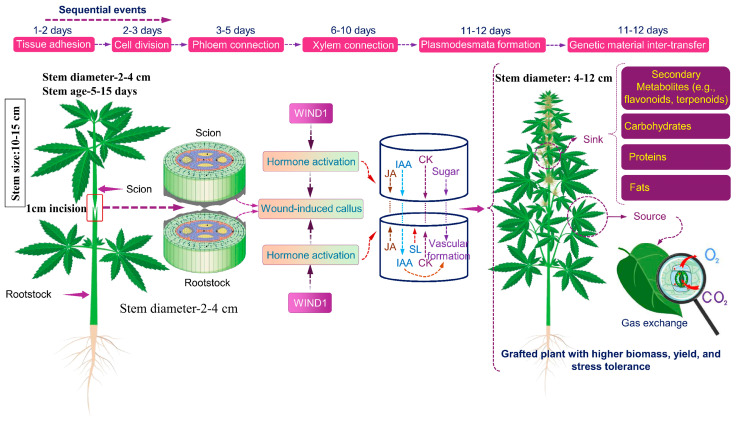
Proposed grafting mechanisms in *Cannabis sativa* for biomass yield and secondary metabolite production. Grafting involves the following sequential stages: tissue adhesion, cell division, callus formation, and vascular reconnection (phloem and xylem). During adhesion, cell wall damage and hormonal signalling (IAA, CK, SL, JA) activated transcription factors (TFs), promoting cell division and vascular cambium activation and forming a callus. WOUND-INDUCED DIFFERENTIATION 1 (WIND1) is a key TF driving callus formation and vascular regeneration by regulating defence, callus development, and vascular reconnection genes. A shared cell wall with plasmodesmata forms between the scion and rootstock, facilitating the transport of proteins, fats, genetic materials, and metabolites. The vascular system enables long-distance communication, while stomata regulate the CO_2_ and O_2_ exchange essential for photosynthesis and carbon accumulation, serving as precursors for primary and secondary metabolites. Abbreviations: IAA, indole-3-acetic acid; CK, cytokinin; SL, strigolactone; JA, jasmonic acid.

The underlying mechanisms driving regeneration capacity in grafted cannabis remain largely unknown [20]. A recent study introduced a single-step grafting methodology for medicinal cannabis, where the freshly cut scion was grafted onto freshly cut donor stems [20]. Two scion varieties with distinct cannabinoid and biomass yields (“CBD1” and “THC2”) but limited root development were grafted onto two high-THCA and low-CBDA rootstocks (“THC9r” and “THC8). This approach altered cannabinoid and biomass yields, demonstrating that grafting can improve yield without extended times [20]. Such methodologies provide valuable insights for investigating root–stem interactions and signalling pathways (Figure 1).

Numerous studies have demonstrated that specific rootstocks enhance plant biomass and yield. Root system architecture plays a critical role in physiological functions, influencing nutrient uptake and overall performance [19]. A recent study examined the effects of three rootstock types, “potentially dwarfing rootstocks (PDR), potentially vigorous rootstocks (PVR), and seedlings-as-rootstocks (SAR)” on graft survival rates, morphological traits, and biochemical composition (cannabinoid and terpene profiles). Surprisingly, PVR and SAR did not outperform PDR across most parameters [19]. Adventitious root formation is primarily influenced by genetic factors and plant age, with additional contributing variables such as leaf area, cutting position, endogenous hormone concentration, light intensity, rooting medium, water availability, and nutrient uptake [19]. Variability in graft survival rates based on rootstock selection suggests that successful rooting alone does not determine graft success. Instead, improvements in growth and yield are likely mediated by rootstock-driven modifications in scion physiology [19] (Table 1).

Understanding the influence of rootstock on shoot growth is critical, as rootstock significantly impacts hormonal dynamics, which are closely linked to plant productivity and resource efficiency. The transport and distribution of auxins and cytokinins likely contribute to biomass production in cannabis grafting. Additionally, stress-induced long-distance signalling molecules may play a role in systemic resistance and secondary metabolism activation [30,31]. Notably, cannabinoids, terpenoids, carotenoids, gibberellins, strigolactone, and abscisic acids share a common biosynthetic precursor, isopentenyl pyrophosphate (IPP) [32]. Studies on rootstock selection in other crops have demonstrated significant improvements in carotenoid profiles, with increased β-carotene and lycopene concentrations [3,5,32]. Given the shared biosynthetic pathways between cannabinoids and terpenoids, it is plausible that grafting may stimulate the production of desirable secondary metabolites in cannabis [4]. Further research should focus on the physiological responses of scions to different rootstocks, alterations in hormone balance, metabolic pathways, and gene expression changes in specific rootstock–scion combinations. Additionally, breeding programmes should prioritize developing rootstocks that are resistant to biotic and abiotic stresses to enhance grafting success and overall plant resistance.

## 4. Grafting: Molecular Background of Rootstock–Scion Joining

Grafting has been used for at least 7000 years. Initially, it was developed for agronomical and ornamental effects, so knowledge remained empirical. We know little about the molecular mechanisms behind rootstock adaptation to different soils and rootstock-conferred scion phenotype changes. Graft union formation and compatibility are poorly understood despite over a hundred years of study [33]. In this process, two individuals must interact efficiently for survival. Integrating tissues involves adult tissues de-differentiating to form new conducting structures. Mechanisms differ significantly for xylem vessels (dead cells) and phloem sieve elements (living cells) [33]. Integration mechanisms remain unclear, and grafting ability is not universal across taxa; dicotyledonous plants graft easily, but monocots cannot, as they lack a vascular cambium [33]. The plant vascular system, comprising the phloem and xylem, is crucial in facilitating communication and transporting organic compounds, hormones, metabolites, proteins, DNA, RNA, water, and nutrients between the shoot and root. Successful vascular reconnection is essential during grafting, as failure in this process can result in long-term graft failure. It is necessary to understand how vasculature reconnection occurs and to monitor connectivity success. Research indicates that the dynamics of graft junction formation are similar, with the common activation of genes associated with procambium, phloem, and xylem, suggesting a conserved mechanism in plant grafting [34,35,36,37,38].

**Figure 2:**
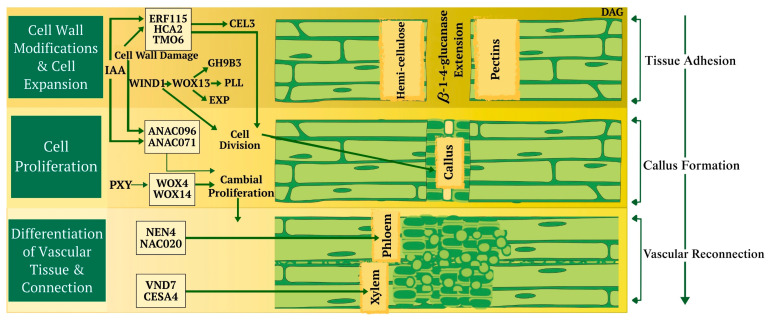
Molecular factors involved in graft formation in plants. Abbreviations: VASCULAR-RELATED NAC-DOMAIN 7—VND7; CELLULOSE SYNTHASE A4—CESA4; NAC45/86-DEPENDENT EXONUCLEASE-DOMAIN PROTEIN 4—NEN4; NAC DOMAIN-CONTAINING PROTEIN20—NAC020; WUSCHEL-LIKE HOMEOBOX4—WOX4; WUSCHEL-LIKE HOMEOBOX4—WOX14; PHLOEM INTERCALATED WITH XYLEM—PXY; Arabidopsis NAC DOMAIN CONTAINING PROTEIN 96—ANAC096; Arabidopsis NAC DOMAIN CONTAINING PROTEIN 71—ANAC071; EXPANSIN—EXP; PECTATE LYASE-LIKE—PLL; WUSCHEL-LIKE HOMEOBOX4-WOX13; GLYCOSYL HYDROLASE 9B3—GH9B3; Indole Acetic Acid—IAA; HIGH CAMBIAL ACTIVITY2—HCA2; ETHYLENE RESPONSE FACTOR115—ERF115; TARGET OF MONOPTEROS 6—TMO6; CELLULASE3—CEL3.

Successful grafting depends on the precise exchange and perception of cellular recognition signals. However, it is generally assumed that the sequence of events occurring at the graft interface is relatively conserved across different species [33,35]. The initial phase of forming a graft union commences with a wound response characterized by the collapse of cells at the graft junction. Within minutes, this triggers electric and ion flux signals, which lead to the accumulation of reactive oxygen species (ROS) and hormones associated with the wound [33,35]. Cellular debris and dead tissue can gather at the graft interface, often forming a necrotic layer, even in homografts. Recent research underscores the necessity of detecting cell wall damage as a fundamental step in triggering wound-healing processes and forming graft unions. Through enzymatic digestion or genetic modification, cell wall changes trigger transcription factors (TFs) that regulate wound responses and the formation of graft union (Figure 2) [33,35]. Effective grafting begins with the grafter making precise cuts and aligning tissues correctly. The plant then starts the process of tissue adhesion through modifications, expansion, the division of cell walls, and crosslinking to connect new cells [34,35,36,37,38,39]. Specific genes, such as those associated with cell division, are activated in both grafting and parasitism, indicating some overlap. Specific genes are involved in the process of wound healing and vascular connection (Figure 2) [34,35].

Disruption in vascular connection results in the accumulation, canalization, and transportation of auxin and photoassimilates above the graft interface; this imbalance might account for the varying cell differentiation rates between the scion and rootstock. Auxin originating from the bud is essential for establishing vascular connections after injury [35,37,40,41] and likely during grafting. Plant hormones, especially auxin, cytokinin, gibberellin, jasmonic acid, and ethylene, are crucial in facilitating the differentiation and regeneration of phloem and xylem tissues [34,42]. In tomatoes, auxin and cytokinin levels reach their peak around the graft junction 12 h post-grafting, with a significant overlap between genes activated by grafting and those responsive to auxin [34,42]. These hormones might play a role in communication between the scion and rootstock above and below the graft interface. Auxin serves as the main regulator of vascular differentiation, with other hormones enhancing its signalling pathway to fine-tune this process [34,43,44,45].

In plants, plasmodesmata are tiny channels that facilitate cell communication by crossing neighbouring cell walls, allowing cytosolic and membrane molecule transfer. These channels originate during cell division by capturing endoplasmic reticulum strands in the new cell wall (primary plasmodesmata) or within existing cell walls through an unknown process (secondary plasmodesmata). In just a few days, secondary plasmodesmata form at the graft junction between the scion and rootstock, facilitating interactions between cells [33,35,40,44]. A set of candidate genes associated with plasmodesmata formation is upregulated at the graft junction during the formation of the graft union [33,35,40,44].

While these interactions often lead to the formation of a viable grafted plant, in many instances, symptoms of physiological distress may emerge during early or later stages of development, ultimately culminating in what is termed “graft incompatibility” [35,46]. The underlying causes of graft incompatibility remain uncertain. It is still debated whether incompatibility arises from inherent partner rejection, differential growth dynamics, or the physiological stress induced by the grafting process itself [47]. A major limitation in understanding graft (in) compatibility is the absence of a universally accepted definition. Broadly, graft compatibility and incompatibility are defined as the respective success or failure in establishing a functional graft [35,46,48]. It is widely recognized that the likelihood of incompatibility increases with greater taxonomic divergence between the scion and rootstock [35,48].

## 5. Implication of Grafting for Crop Improvement: Prospects for *Cannabis sativa*: Yield and Secondary Metabolite Production

The concept that the graft interface might isolate or change the transmission of signals between the scion and rootstock is intriguing. Numerous small RNAs present in phloem sap can be transmitted through grafting [33,34], indicating their potential to influence rootstock–scion communication. Interspecific grafting can alter DNA methylation patterns, which may be the basis for traits conferred by rootstock in crop species [33,34]. Grafting serves as a valuable tool for horticulturists to enhance plant characteristics, boost disease resistance, and achieve higher yields [33,34]. Although many successful grafts between different species and genera exist, our comprehension of what makes grafting successful remains incomplete.

Grafting can enhance horticultural output, help identify rootstock and scion genotypes, and possibly lead to the creation of new species through horizontal gene transfer. The differential expression of genes during grafting has been explored, along with their challenges and novel applications in agriculture [35,43]. The movement of sugars, fats, minerals, proteins, hormones, peptides, messenger RNA (mRNA), and small interfering siRNA across the graft junction is becoming recognized as a crucial mechanism in regulating rootstock–scion communication and grafting physiology [35,43]. Grafting enhances the cultivation of fruit, vegetables, and medicinal crops by improving adaptability and stress resistance. The success of grafting relies on the proper alignment of the anatomical structures of the rootstock and scion; the misalignment of vascular elements can result in graft failure [35,43]. Heterografting impacts essential plant processes such as water uptake, nutrient absorption, hormonal signalling, and enzyme activity.

In plants, as in other eukaryotic organisms, the vertical transmission of genetic information through reproduction plays a fundamental role in preserving species integrity [3,5,32]. However, numerous studies in recent years have clearly demonstrated that horizontal gene transfer (HGT)—the interspecific exchange of genetic material across reproductive barriers—is widespread in nature and occurs across all domains of life, including plants. Historically, HGT in multicellular organisms has been regarded as a rare and insignificant phenomenon with minimal evolutionary impact [3,5,32]. However, advances in next-generation sequencing (NGS) technologies have revolutionized our ability to detect HGT events through comparative genomic analyses, significantly increasing their documented occurrence [3,5,32]. Grafting has emerged as a powerful experimental system demonstrating that not only hormones and metabolites but also genetic materials such as messenger RNAs, small RNAs, and even fragments of genomic DNA can move between grafted partners via vascular connections [37,49,50,51,52].

The observation that this genetic material exchange may occur through localized cellular interactions rather than long-distance vascular transport suggests the key role played by the plasmodesmata rather than the phloem or xylem. Furthermore, instances of HGT between rootstock and scion, along with siRNA-mediated epigenetic modifications that are transmissible through grafting, could potentially explain the heritable phenotypic variations observed post-grafting, although robust experimental confirmation is still needed [37,49,50,51,52]. Evidence from heterografting experiments also supports the systemic movement of transcripts or RNA–protein complexes over long distances, which are believed to play critical regulatory roles in plant development and physiological responses [37,49,50,51,52].

Gene technology regulators receive applications seeking permission for the environmental release of genetically modified (GM) plants, many of which possess beneficial traits such as improved production, enhanced nutrition, and resistance to drought, pests, and diseases [53]. Gene transfer through HGT also needs to be considered as part of the risk assessment [54]. The likelihood of the HGT of the introduced or transgenic DNA from GM plants to other organisms depends on its proportion in relation to the total amount of plant DNA [55,56]. Many adverse effects/risks are due to the potential of the HGT of the transgene from GM plants to other plants [57]. Therefore, these potential effects should be evaluated on a case-by-case basis in the context of the proposed activities in the risk assessment of each GM plant if the occurrence of HGT is considered more likely than when dealing with a non-GM plant. The advances in whole genome sequencing and comparative genomics can predict HGT events in future cannabis plants. However, up until now, there have been no reports of adverse impacts/risks on pharmaceutical cannabis as a direct or indirect result of HGT from GM plants or non-GM-grafted plants.

RNA interference (RNAi) is a conserved biological mechanism found in nearly all eukaryotic organisms. RNAi-based genetically modified organisms (GMOs) have emerged as powerful tools in plant breeding, owing to their ability to regulate gene expression with high-sequence specificity. This technology offers numerous agricultural applications, including enhanced resistance to biotic and abiotic stresses, improved nutritional and industrial traits, delayed fruit ripening, the induction of male sterility, modification of plant architecture, and elimination of allergens and toxic compounds [58,59]. The ability of RNAs to move across the graft interface has opened up new avenues for genetic engineering [60]. Thousands of mRNAs are known to travel systemically between shoots and roots, and many of these mobile transcripts contain a tRNA-like structure (TLS) that facilitates their movement [60]. By fusing a TLS to both Cas9 mRNA and CRISPR guide RNAs, researchers have developed a mobile CRISPR-Cas9 genome editing system. In this approach, grafting allows the transgene-derived RNAs to be transported from a genetically modified rootstock to a wild-type scion, resulting in heritable genome modifications in the scion’s progeny [60]. This strategy holds great promise for generating transgene-free CRISPR-Cas9 edits. However, further research is necessary to refine and expand its applicability. Establishing a reliable mobile RNA-based delivery system via grafting may represent a significant technological advancement for genome editing across diverse plant species [60]. Indeed, both RNA interference (RNAi) and CRISPR/Cas technologies hold transformative potential for cannabis research and breeding [58,59,61,62].

To enhance risk assessment in genome editing, off-target prediction assays are routinely employed to improve the safety and precision of these technologies [62]. The development of effective and safe genome editing strategies typically involves a combination of in silico or in vitro cell-free DNA-based off-target prediction assays, followed by cellular and genome-wide validation techniques [62]. These safety evaluation methods have been designed to identify potential off-target sites; detect structural variations such as chromosomal translocations, chromothripsis, and aneuploidy; and assess unintended editing outcomes and epigenetic alterations [62]. Significant progress has been made in optimizing CRISPR/Cas systems to enhance on-target efficiency and specificity while minimizing off-target activity and cytotoxic effects in plants [59]. Key technical variables influencing editing outcomes in plants include the design of guide RNAs (gRNAs), the selection of Cas protein variants, the format and composition of CRISPR components, and the method of delivery, particularly those utilizing nanomaterials or viral vectors for introducing CRISPR/Cas reagents into the genomes of grafted plants [59,60]. Integrating advanced approaches such as the use of chimeras, interspecies hybrids, and mobile RNAs with emerging grafting technologies may revolutionize the field of graft biology [35,61]. These innovations hold significant promise for creating novel species and transformative biotechnological tools. Given the rapid advancements and growing research interest over the past two decades, the future of grafting research appears highly promising, particularly in advancing the development and genetic improvement of *Cannabis sativa*.

Molecular genetic research using *Arabidopsis thaliana* has uncovered the molecular mechanisms behind floral induction in plants [63]. The main genetic routes that play a role in flowering are the photoperiod, vernalization, autonomous, and gibberellin pathways. New flowering pathways have been identified, including those related to age, thermosensory, sugar, stress, and hormonal signals [63]. Each hormonal signal can be analyzed in terms of signal transmission, but comprehensive models for the flow of each phytohormonal signal to regulate flowering have yet to be established [63].

Although its application in horticulture and science is on the rise, the intricacies of plant grafting remain somewhat mysterious, with eight key plant hormones being essential for wound repair and vascular development [36]. Through grafting, it was discovered that the mobile protein FLOWERING LOCUS T (FT), which promotes flowering, is produced in leaves and then moves to the plant’s meristems [12]. Grafting-induced flowering is a critical phenomenon for understanding systemic floral induction mediated by florigen. Beyond its biological significance, this mechanism also serves as a valuable breeding strategy, particularly for accelerating seed production in crop species with extended generation times [64]. The FLOWERING LOCUS T (FT) protein, a principal component of florigen, has been shown to play a pivotal role in this process. Studies using intergeneric cabbage/radish grafts demonstrated that the extent of floral induction in the scion is quantitatively associated with the level of FT protein accumulation, indicating that changes in FT levels directly influence the flowering response in grafted plants [64].

This principle involves grafting a plant lacking a particular substance to one that possesses it; if the substance is detected in the previously deficient plant after grafting, it indicates mobility. Grafting rootstocks that lack siRNAs or siRNA-rich scions results in the restoration of siRNAs in the rootstocks [12]. Additionally, grafting has demonstrated the mobility of phytohormones. Before strigolactones were identified, grafting with branching mutants suggested the presence of a graft-transmissible hormone that inhibited branching, which was later identified as a strigolactone [34]. Similarly, grafting revealed the mobility of GA12, a precursor to gibberellic acid [34].

The complex interactions among phytohormones, mediated by key regulators, have been extensively explored to combat abiotic and biotic stress [28]. Throughout their life cycle, plants encounter various environmental stresses. To adapt and thrive, they have developed defence strategies involving secondary metabolites (SMs), which enhance their resilience. These bioactive compounds deter herbivores, serve as barriers against pathogens, and protect against oxidative stress [30,65]. Plants modulate the synthesis and accumulation of SMs in response to environmental cues and hormonal signals [30,65]. Transcription factors (TFs) play a vital role in this process by regulating the gene expressions necessary for SM biosynthesis. Different hormone-responsive TFs, such as “AP2/ERF, WRKY, bHLH, bZIP, MYB, and NAC”, fine-tune defence mechanisms to ensure timely SM accumulation, mitigating the effects of stress [30,65]. Grafting can be used to study hormone-induced secondary metabolism and flowering in *Cannabis sativa*. The mobility across junctions mediated by plasmodesmata offers new opportunities to utilize grafting for genetic engineering [2,61,66,67].

Grafting serves as a bridge between science and horticulture. Machine learning-mediated multi-omics integration holds promise for understanding cannabinoid biosynthesis pathways [2]. The use of genome editing tools in cannabis research has shed light on the pathways responsible for cannabinoid and terpenoid biosynthesis, as well as the essential transcription factors involved in trichome development and cannabinoid production [2,61,66,67]. Gene-editing technologies, including “ZFNs, TALENs, and CRISPR-Cas systems”, offer promising opportunities to modify biosynthetic pathways, thereby enhancing enzyme efficiency and facilitating the development of novel cannabis traits [2,61,66,67]. NMR-based metabolomics has proven effective in directly evaluating the metabolic and physiological states of trichomes and/or living cells, as well as observing shifts in cannabinoid concentrations and profiles due to environmental influences [2,61,66,67].

## 6. Conclusions

Grafting is used to improve plant quality and yield. The rootstock significantly influences the scion’s growth, development, flowering, fruit set, and stress resilience. Rootstock and scion interactions and their molecular regulation are complex processes. Healing between the rootstock and scion facilitates compound exchange, phytohormone regulation, gene expression, and the long-distance transport of genetic materials, nutrients, and water, thus aiding in crop quality, production, stress resistance, and quicker breeding. Graft hybrids at the junction can be used for asexual hybridizing species like hemp. Grafting research has a robust prospect with new potential combinations and applications. Recent advances in silencing transmissible RNA and mobile CRISPR-Cas9 editing across the junction have offered new ways to genetically engineer plants. Moreover, hormonal signalling occurs at this interface during the merging process of the scion and rootstock. Future research should focus on hormone interactions, biomass production, and secondary metabolite biosynthesis in cannabis. Incorporating different omics technologies can significantly enhance our understanding of hormone-induced growth and metabolite synthesis pathways, facilitating the identification of enzymes or variants for higher secondary metabolite productivity in cannabis. Grafting represents a relatively novel cultivation technique in *Cannabis* production and necessitates additional resources, including specialized equipment, adequate space, extended time, and skilled personnel.

## Figures and Tables

**Table 1:** Different rootstock–scion combinations and their effects on biomass production and secondary metabolism.

SL.	Scion Genotype	Rootstock Type	Effect on Biomass Accumulation	Effect on Metabolic Traits	References
**1.**	High-THC cultivar, “Freud Super-Ego” (FSE)	Three chemotypes: high-THC (T), high-CBD (C), and balanced chemotypes (B)	Grafting significantly enhanced the stem girth of the FSE scion across all rootstock types. Leaf mineral contents were markedly elevated in grafted plants. A positive correlation was established between root biomass and stem diameter. Inflorescence yield was substantially higher in all grafted plants relative to non-grafted controls. No evidence of yield suppression was observed in composite plants.	Grafting treatments consistently elevated THC concentrations in FSE’s inflorescences by 8–12%.	[7]
**2.**	CBD1-high CBDA, low-yielding variety. THC2-high THCA, high-yielding line with unstable root development.	“THC9r” and “THC8r”: both high-THCA, low-CBDA genotypes.	Biomass production in THC2 scion grafted onto THC9r rootstocks increased by 20% compared to non-grafted THC2. Notable improvements in plant architecture and biomass yield were recorded in grafted treatments.	In CBD1-grafted plants, CBDA levels and concentrations of certain minor cannabinoids differed significantly from those of non-grafted counterparts. In THC9r_2s-grafted plants, an elevated THCA concentration combined with increased biomass led to an enhanced THCA yield from 8 to 13 g Plant^−1^.	[20]
**3.**	“ScionII”—chemotype II (industrial hemp) “ScionIII”—chemotype III (high-CBD type)	Three rootstock categories: PDR—potentially dwarfing rootstocks PVR—potentially vigorous rootstocks SAR—seedlings as rootstocks	Rootstock type significantly influenced the bud compactness index in “ScionII” when grafted onto PDR. Comparative assessment revealed that grafted combinations exhibited superior performance across multiple agronomic traits relative to non-grafted scions.	Grafting ScionIII onto PDRs resulted in a 27% increase in CBD concentration. The CBD yield per plant increased by 71% and 84%, respectively, in ScionII when grafted onto SAR. SARs were identified as the most efficient rootstocks for maximizing CBD productivity.	[19]
